# 

**Published:** 1922-05

**Authors:** 


### RECENT APPOINTMENTS.

### Institutional.

Dr. Bride M. Martyn, to be Medical Superintendent of Holgate and Bloomlands Hospitals, Middlesbrough.

Mr. T. H. W. Hicks has resigned the position of secretary to the Darlington Hospital and Dispensary. His successor is Mr. Riddle.

Mr. G. Swift has been elected chairman of the Evesham Hospital in succession to Mr. Geoffrey New, who has retired after two years' exacting work.

Dr. A. H. Macpherson, late of Royal Victoria Hospital Farm Colony, Polton, and Hairmyres Sanatorium and Colony, Lanarkshire, to be Resident Medical Superintendent for Burrow Hill Sanatorium.

### Public Health.

Mr. D. C. Lamont, M.B., Ch.B., D.P.H., late House Physician, Fever Wards, Edinburgh City Hospital, to be Assistant Tuberculosis Officer for the County Borough of Stoke-on-Trent.

Mr. H. G. Donald, M.B., Ch.B., D.P.H., late Assistant School Medical Officer for the North Riding of Yorkshire, and Mr. James A. Stirling, M.B., Ch.B., D.P.H., late Deputy Medical Officer of Health, School Medical Officer and Joint Tuberculosis Officer for Kincardine, to be Assistant School Medical Officers to the County Council of Durham.

Mr. James Todesco, late House Surgeon at Sheffield Royal Infirmary, to be Resident Medical Superintendent of the Borough Hospital, and Assistant Medical Officer of Health for the County Boroughof Croydon.

Mr. Charles L. Sutherland, M.B., Ch.B., D.P.H., late District Tuberculosis Officer for the West Riding C.C., to be Assistant Medical Officer of Health for the County Borough of Stockport.

Miss Katherine M. L. Gamgee, M.R.C.S., L.R.C.P. (Lond.), late Demonstrator in Physiology at King's College for Women, to be Assistant Medical Officer (Maternity and Child Welfare) for the City and County of Kingston-upon-Hull, from June 1.

Dr. William Baxter, late R.A.M.C., to be Medical Officer of Health and School Medical Officer for the Borough of Newark-on-Trent, and Medical Officer of Health for Newark R.D.C., Claypole D.C., and Southwell D.C.

Dr. N. M. Donnelly, M.B., B.Ch., D.P.H., late Tuberculosis Officer and Assistant M.O.H. for Deptford, to be Tuberculosis Officer for St. Pancras.

Mr. G. D. E. Jones, late surgical specialist at Woolwich Royal Arsenal, to be Medical Officer for the East Plumstead Relief District.

Mr. Leonard Hearn, late Asst. R.M.O., Royal Victoria Infirmary, Newcastle-on-Tyne, to be Assistant Tuberculosis Officer for the City and County of Bristol.

### Managerial Notices.

*All letters on Business matters must be addressed to the Manager, "The Hospital and Health Review," 28 and 29, Southampton Street, London, W.C. 2.*

**It is especially requested that remittances be made by Cheque or Postal Order and not Stamps.**

RATES FOR SUBSCRIPTION (payable in advance).

| | |
|---|---|
| Three Months (including Postage) ... ... ... | 2s. 0d. |
| Six Months do. ... ... ... | 3s. 9d. |
| Twelve Months do. ... ... ... | 7s. 6d. |

Should the cost of printing and paper, as well as increased postage, necessitate alteration of these rates, the period paid for will be reduced proportionately on unexpired subscriptions.

Subscriptions, which may commence at any time, are payable in advance. Cheques and Post-Office Orders should be crossed London County and Westminster Bank, Covent Garden Branch, and made payable to the Manager of THE HOSPITAL AND HEALTH REVIEW.

*SUBSCRIPTION FORM.*

*Please send me* THE HOSPITAL AND HEALTH REVIEW *for*..................*months, commencing with your issue of* ....................*, for which please find enclosed*..................

*Name* ..................................................

*Address* ..................................................

..................................................

..................................................

*Date* ..................................................

*To* .................................................. *Newsagent.*

### PARLIAMENTARY QUESTIONS AND ANSWERS.

April 3.—Sir A. Mond, answering Mr. T. Griffiths, stated that he had intimated to the chairman of the General Nursing Council his willingness to approve such rules as the Council thought necessary to speed up registration and secure an adequate electorate within the time allowed by the Act. He demurred in the strongest manner to any suggestion that any one section of the Council was less anxious than another to do what is best in the interest of the working nurse.

April 5.—Sir A. Mond informed Mr. Gilbert that he had not issued instructions to medical officers in poor law or other institutions to use the Schick treatment for the prevention of diphtheria.

April 6.—Sir A. Mond assured Mr. T. Thomson that in considering amendments to the Milk and Dairies Act, 1915, he would not overlook the question of giving local authorities power to classify milk, to revoke or suspend certificates, and to register all milk sellers in their area.

April 12.—Sir A. Mond stated, in reply to Mr. G. Barker, that in present financial conditions he could hold out no hope of reconsidering the decision that no part of the Exchequer grant to the voluntary hospitals should go towards capital expenditure

In reply to Mr. Richardson, Sir A. Mond said that it seemed to him that the nurse members of the General Nursing Council were fairly represented on the new committees of the Council, and that he did not propose to make representations to the Council on a matter which was wholly within their discretion.

Sir A. Mond informed Sir A. Holbrook that a bill dealing with smoke-abatement was being prepared, but that he could not yet say whether it would be possible to find time for its introduction.

### NEW BILLS AND LEGISLATIVE PROPOSALS.

March 29.—The National Health Insurance Bill, 1922, was read a first time. This bill transfers to the funds of Approved Societies that part of the cost of medical benefit (apart from the statutory two-ninths specified in the original Act of 1911) which has hitherto been met out of an Exchequer Grant. This transfer is to operate until December 31, 1923, after which date presumably the remuneration of insurance doctors will be again reviewed.

April 5.—The Audit (Local Authorities, &c.) Bill was read a second time in the House of Commons. This bill substitutes a yearly for a half-yearly audit in the case of all local authorities, except London poor-law authorities, and also enables municipal corporations to adopt the system of Government audit by passing a resolution to that effect.

April 7.—The Local Government and Other Officers' Superannuation Bill, introduced by Sir H. Nield, was read a second time. The bill is a permissive one only, and Sir A. Mond, while not opposing it, drew attention to several points which would have to be carefully considered in committee.

April 12.—The Royal Assent was given to the Unemployment Insurance Bill. While the bill was in committee in the House of Commons an amendment was proposed by Mr. Lyle, and accepted by Dr. Macnamara, providing that from July 1 next female professional nurses for the sick, and female probationer nurses, shall no longer be compulsorily insurable under the principal Act. A proposal to limit the exemption to registered nurses was withdrawn. Nurses will continue to be insurable under the Health Insurance Acts.

(*Parliament adjourned on April 12 for the Easter Recess and meets again on April 26.*)

### KING EDWARD'S HOSPITAL FUND: SCHEME FOR WAGE-EARNERS.

The main lines of the following contributory scheme for the London hospitals have been accepted by the General Council of King Edward's Hospital Fund.

### 1.—Objects of the Scheme.

To enable wage-earners, by means of an organised system of moderate and continuous contributions :—

(i) To give voluntary assistance to the hospitals of London in carrying on their work of curing disease and alleviating suffering; and (ii) to be relieved, together with their dependents, from all hospital charges when receiving hospital services.

### 2.—Need for the Scheme.

The expenditure of the hospitals has grown enormously in recent years, partly owing to the rise in wages and in the price of provisions, fuel, &c., and partly owing to the costliness of the equipment needed to fight disease by modern methods. The result is that many of the hospitals are overwhelmed with debt, and in some cases they have had to close many beds. While they will continue, in accordance with the principles on which they were founded, to care for the necessitous poor without payment, they can no longer exempt from payment persons in recept of good regular wages or income. Many hospitals are already making charges towards the cost of maintenance, but such charges represent only a fraction of the cost, and even so are frequently too heavy for patients to pay when incapacitated by illness. It is only reasonable therefore, that the classes from which the majority of hospital patients are drawn should save while in health for the hospitals, and that the savings should be pooled, so that when a contributor has been admitted to hospital treatment a fund may be available to lighten the burden of the hospital.

### 3.—Scope of the Scheme.

It is not intended that hospitals should afford any facilities other than those already provided by them, or that any alteration should be made in the arrangements now in force at individual hospitals for the admission of persons as in-patients or out-patients, or for their treatment by the medical staffs; the hospital will continue to decide what cases are suitable for hospital treatment and the order of priority in which applicants are to be admitted. The scheme has nothing to do with National Health Insurance, and leaves untouched the position and responsibility of the general practitioner; it does not cover ordinary maternity cases or any treatment for which provision is made by the State or local authorities.

### 4.—Who May Join the Scheme.

In order to avoid abuse by those who can afford to pay for treatment outside the hospital, it is necessary to fix a strict income limit. Accordingly, the scheme is confined to persons whose individual income does not exceed £4 a week for a single man or woman, £5 a week for a man and wife with no children under 16, £6 a week for a man and wife with children under 16. Such persons being employees in a factory or other unit of employment, or members of societies established for mutual benefit, will join in groups.

### 5.—Organisation.

All contributions will be collected within the factory or other group, and will be paid over to a central organisation distinct from the King's Fund, and from any individual hospital, but so composed as to secure the co-operation of all interests concerned. This organisation, unless and until hospitals otherwise arrange, will distribute the funds collected under the scheme, after deduction of expenses of collection and administration, as payment towards the cost of patients after admission to hospital.

### 6.—Condition.

Regular contributors of 12s. per annum paid in one sum in advance, or 13s. per annum paid in weekly instalments of 3d., will, together with their wives and children under 16, when accepted for treatment by a hospital, be relieved, by payments made on their behalf by the central organisation, from contributing to their cost of maintenance as in-patients and from any charge in all out-patient departments.

### H.M. STATIONERY OFFICE PUBLICATIONS.

[The prices quoted include postage.]

Estimates for Civil Services for the year ending March 31. 1923. Class VII. Health, Labour and Insurance, (H.C. 32-VII.). 1s. 1½d.

House of Lords Papers and Bills: (42). Lunacy. 3d.

House of Commons Bills: (73). National Health Insurance. 4d.

Atmospheric Pollution. Report on Observations in the year ended March 31, 1921. 2s. 1½d.

National Insurance (Unemployment) Acts, 1911-1918. Unemployment Fund Account, July 13, 1919, to July 17, 1920, with the Report of the Comptroller and Auditor-General thereon. (H. C. Paper 34). 3d.

National Health Insurance Fund Accounts for 1919 (England, Wales, Scotland and Ireland), with the Report of the Comptroller and Auditor-General thereon. (H. C. 22). 2s. 1½d.

National Health Insurance: (281). Arrears Amendment Regns., 1922. (282). Calculation of Contributions Regns., 1922. 2d. each.

National Health Insurance Act, 1911-1921. 8th Report on the work of the N.H.I. Audit Dept., 1921. 10d.

Command Papers: (1631). National Health Insurance Joint Committee. Financial Provisions of N.H.I. Bill, 1922. 3d.

Quarterly Return of Births, Deaths and Marriages registered in the Divisions, Counties and Public Health Districts of Scotland, to Dec. 31, 1921. 7d.

Ministry of Health Circular, No. 289, to Local Authorities, March 14. 2d.

Ministry of Health Provisional Order, No. 1. (H. C. B. 54). 7d.

Ministry of Health Provisional Orders (Leeds and Bradford Extension). H. C. B. 53. 4s. 2½d.

Ministry of Health Provisional Order (Guildford Extension). H. L. B. 29. 7d.

Ministry of Health Provisional Order (Tamworth Extension). H. L. B. 30. 1s. 1½d.

Laws and Regulations relating to Lead Poisoning. An analysis with texts of the laws and regulations made in the chief industrial countries to prevent Plumbism, by Gilbert Stone. 5s. 4½d.

No. 299. Sanitary Officers' Order. To County Councils, Metropolitan Councils, Town, Urban and Rural District Councils, Port Sanitary Authorities. (E. and W.) 2d.

Hospital Exemption No. 1 Order, 1922. 2d.

No. 276. Medical Officer of Health and Sanitary Inspector, England and Wales. 3d.

Meat Inspection, Memorandum on a system of, recommended by the Ministry of Health for adoption by Local Authorities and their Officers. 2d.

Experimental Cottages. A Report on the work of the Department of Scientific and Industrial Research at Amesbury, Wilts. 5s. 3d.

### Statutory Rules and Orders.

Draft. National Health Insurance. Termination of Sanatorium Benefit Amendment Regulations, 1922. Draft dated March 10, 1922, of regulations proposed to be made under Section 4 of the N. H. I. Act, 1920. 2d.

Draft. National Health Insurance. Provisional Regulations dated March 8, 1922, under Section 4 of the N. H. I. Act, 1920, amending the N. H. I. (Termination of Sanatorium Benefit) Regulations, 1921. 2d.

No. 217. National Health Insurance (Medical Benefit) Amendment Regulations dated March 9, 1922. 2d.

No. 241/S. 17. Housing, Scotland. Local Authorities (Assisted Housing Schemes) Amendment Regulations (Scotland), dated March 18, 1922, made by Scottish Board of Health. 2d.

Draft dated March 17, 1922, of Regulations proposed to be made by the National Health Insurance Joint Committee and the Minister of Health and the Scottish Board of Health amending the N. H. I. (Deposit Contributors) Regulations, 1919-20. 2d.

Ministry of Health. Byelaws (Draft form): New Streets and Buildings. 1s. 1½d.

Memorandum on the effect of the Summer Time Act on the Health of School Children. (Cmd. 1622). 4d.

### OFFICIAL REPORTS RECEIVED.

Weekly Reports of the United States Public Health Service. (Government Printing Office, Washington.) Vol. 37.

Reprints from the United States Public Health Reports:—

No. 682.—The Work of the Public Health Service in the Care of Disabled Veterans of the World War.

No. 687.—Smallpox in Twenty States, 1915-1920.

No. 692.—Variations in Case Fatality during the Influenza Epidemic of 1918.

No. 704.—The Care of Neuro-Psychiatric Disabilities.

No. 709.—A Note on the Natural Immunity of Wild Rats to Plague.

Warrington: Annual Report to the Education Authority on School Hygiene, 1921. By Dr. G. W. N. Joseph, M.D., D.P.H., Medical Officer of Health and School Medical Officer.

The Nurses' Co-operation, late of 8 Cavendish Street, W., now of 22 Langham Street, Portland Place, W. (1921).

County Borough of Eastbourne Local Education Authority: Medical Officer's Report, 1921.

Council of the County Palatine of Durham: Annual Report of the Medical Officer of Health, and other Records for 1919.

Derbyshire Branch of the British Red Cross Society, 1921.

Leicester and County Saturday Hospital Society. Report and Summary of Receipts and Payments, 1921.

**Hospitals and Homes :—**

Royal Devon and Exeter Hospital, and Ockenden Convalescent Home, Torquay, 1921.

Royal Portsmouth, Portsea and Gosport Hospital, 1921.

Gravesend Hospital, Kent, 1921.

Chesterfield and North Derbyshire Royal Hospital, 1921.

General Hospital, Birmingham, 1921.

Blackburn and East Lancashire Royal Infirmary, 1921.

The Royal Northern (Group of) Hospitals, 1921.

Preston and County of Lancaster Queen Victoria Royal Infirmary, 1921.

Norfolk and Norwich Hospital, Norwich, and Fletcher Convalescent Home, Cromer, 1921.

Clackmannan County Hospital, 1921.

Hospital for Consumption, Brompton, 1921.

General Hospital, Nottingham, 1921.

Walsall General Hospital, 1921.

Mildmay Memorial Hospital, Newington Green Road, N.1, 1921.

Infants' Hospital, Vincent Square, Westminster, S.W.1, 1921.

St. Bartholomew's Hospital, Rochester, 1921.

St. George's Hospital and Atkinson Morley's Convalescent Hospital, 1921.

The Meath Home of Comfort for Epileptics, Westbrook, Godalming, 1921.

### MEMORANDA FOR THE MONTH.

A ball in aid of the Victoria Hospital for Children, Chelsea, will take place at Claridge's on May 8. Princess Victoria has given her patronage.

The Dental Board of the United Kingdom will begin its next session on May 9 at 2 p.m., when the chairman, Mr. F. D. Acland, M.P., will give an address.

The People's League of Health has arranged a series of six lectures on "The Economy of Health and the Human Side of Industry and Commerce," to be held at the Royal Society of Arts, beginning on April 26 at 6 p.m. Tickets may be obtained from the honorary organiser, Miss Olga Nethersole, the People's League of Health, 12 Stratford Place, Oxford Street, W.1.

As we go to press, Prof. Sir Arthur Keith and Prof. Shattock are beginning a course of demonstrations of specimens in the Museum of the Royal College of Surgeons. The remaining demonstrations, which are open to advanced students and medical practitioners, will be given in the theatre of the College in Lincoln's Inn Fields at 5 p.m. on April 28 and May 1, 5 and 8.

